# Incentive payments are not related to expected health gain in the pay for performance scheme for UK primary care: cross-sectional analysis

**DOI:** 10.1186/1472-6963-12-94

**Published:** 2012-04-16

**Authors:** Robert Fleetcroft, Nicholas Steel, Richard Cookson, Simon Walker, Amanda Howe

**Affiliations:** 1 Department of Population Health and Primary Care, Norwich Medical School, University of East Anglia, Norwich, UK; 2 Centre for Health Economics, University of York, York, UK

**Keywords:** Physician incentive plans, Primary care, Quality indicators

## Abstract

**Background:**

The General Medical Services primary care contract for the United Kingdom financially rewards performance in 19 clinical areas, through the Quality and Outcomes Framework. Little is known about how best to determine the size of financial incentives in pay for performance schemes. Our aim was to test the hypothesis that performance indicators with larger population health benefits receive larger financial incentives.

**Methods:**

We performed cross sectional analyses to quantify associations between the size of financial incentives and expected health gain in the 2004 and 2006 versions of the Quality and Outcomes Framework. We used non-parametric two-sided Spearman rank correlation tests. Health gain was measured in expected lives saved in one year and in quality adjusted life years. For each quality indicator in an average sized general practice we tested for associations first, between the marginal increase in payment and the health gain resulting from a one percent point improvement in performance and second, between total payment and the health gain at the performance threshold for maximum payment.

**Results:**

Evidence for lives saved or quality adjusted life years gained was found for 28 indicators accounting for 41% of the total incentive payments. No statistically significant associations were found between the expected health gain and incentive gained from a marginal 1% increase in performance in either the 2004 or 2006 version of the Quality and Outcomes Framework. In addition no associations were found between the size of financial payment for achievement of an indicator and the expected health gain at the performance threshold for maximum payment measured in lives saved or quality adjusted life years.

**Conclusions:**

In this subgroup of indicators the financial incentives were not aligned to maximise health gain. This disconnection between incentive and expected health gain risks supporting clinical activities that are only marginally effective, at the expense of more effective activities receiving lower incentives. When designing pay for performance programmes decisions about the size of the financial incentive attached to an indicator should be informed by information on the health gain to be expected from that indicator.

## Background

The 2004 General Medical Services (GMS) contract for UK primary care represented a major shift in funding towards the use of pay for performance to incentivise quality improvement [1]. The UK contract is currently the world's largest experiment in pay for performance in primary care, currently costing £1 billion (€1.14 billion) a year or 15% of spend on primary medical care in England alone [2]. Similar developments are happening in other countries including the USA [3], Canada [4], Australia [5], New Zealand [6], Germany [7], Netherlands [8], and Spain [9]. Although the GMS contract rewards general practitioners for the level of performance, its main aim was to improve the quality of care [2]. The pay for performance element of the GMS contract was introduced in 2004 and revised in 2006, and is known as the Quality and Outcomes Framework (QOF). The intention of the GMS contract was to reward 'GPs and their staff for the volume and quality of the work done' [1].

There were 76 clinical indicators in the 2004 version of the GMS contract with 550 financial points, and an additional 11 points for performing cervical screening [1]. There were 80 clinical indicators in the 2006 revisions of the GMS contract accounting for 655 financial points. Each point was worth £75 (€85) in 2004 rising to £124.60 (€141) from 2005 for an average sized English practice comprising of 6411 patients and 3 full time GPs. Points are awarded in proportion to the achieved level of the indicator between a lower and upper limit of payment thresholds. These payment thresholds start at a minimum activity of indicated care of 25-40% rising to a maximum payment for 60-90% of activity. Full incentive payment is received before all patients have received treatment for 2 reasons. First, patients who are considered unsuitable for treatment can be exception reported. Exception reporting is the exclusion by their doctor of patients from receiving a specific intervention because they have been deemed not suitable for that intervention. Second, performance thresholds for maximum payment are set below 100% [1].

The introduction of the QOF led to a substantial rise of around 25% in GP principal's incomes [10], with general practices achieving an average of 96% of available QOF points [11]. Critics have argued that the QOF represented poor value for money [12] and, in particular, failed to apply the same rigorous cost-effectiveness test applied elsewhere in the NHS by the National Institute for Health and Clinical Excellence (NICE) [13,14]. Partly in response to these criticisms, the UK government has recently introduced a new process for revising the QOF, which is informed by cost-effectiveness evidence produced by NICE [2]. This idea is not new, and linkage between the selection of performance indicators for primary care and their potential health gain was first suggested in 1992 [15]. An explicit motivation for this process was that 'QOF indicators should be more focused on health outcomes and delivering health improvement with rewards aligned to the overall health need or health benefit' [2]. This policy change of focusing on health outcomes (rather than processes of care) has also been suggested by other authors [16]. However, this new process for revising QOF only goes part of the way towards addressing cost-effectiveness concerns, since it focuses on the selection of QOF indicators and not on the size of the financial incentives applied to different indicators. It is important that incentives are selected and weighted appropriately because incentives have been shown to change practice, and areas of care not receiving incentives may be marginalised [17,18]. There is concern over the effectiveness of P4P schemes where sustained quality improvement may differ little from the underlying trend [19,20].

The only previous study of the size of incentives and expected health outcomes in pay for performance schemes found that payments did not reflect likely health gain [13]. This was a small scale study of 6 interventions in the 2004 version of QOF, and did not include quality adjusted life years (QALYs) as an outcome. In our paper, we examine whether the financial incentives currently applied to different QOF indicators are likely to be appropriate from a cost-effectiveness perspective. Cost effectiveness principles do not necessarily require a linear relationship between incentive payment and health gain, for two reasons. First, there may be a non-linear relationship between the size of health gains and the size of treatment costs, which also have to be taken into account in the cost-effectiveness calculus. Second, there may be a non-linear relationship between the size of incentive payments and the probability the indicator will be achieved. Nevertheless, it is reasonable to suppose that there should be a positive albeit non-linear relationship between pay and performance in terms of health gain, if cost effectiveness principles are being appropriately applied.

Our objective was to test in two ways the simple proposition that performance indicators with larger population health benefits receive larger financial incentives. The first way was to examine the association between the marginal health gained and additional incentive received for a 1% point increase in indicator performance in a practice operating between the minimum and maximum thresholds for incentive payments. The second way was to examine the association between the maximum incentive payments and expected health gain from performance at the threshold for receiving that maximum incentive payment. We examined these associations focusing on the incentives faced by an average sized general practice in England with three full time practitioners and a list size of 6411.

## Methods

We performed a cross sectional analysis of the association between the size of financial incentives and expected health gain in English primary care in the 2004 and 2006 versions of the Quality and Outcomes Framework in the following manner.

### Data Collection

We obtained information on clinical indicators and incentive payments for practices from the GMS contract documentation. We obtained data on disease prevalence and on exception reporting for English practices from the NHS Information Centre [11]. We obtained data on estimated health gain in terms of lives saved and QALYs using published estimates of the likely maximum expected health gains for 28 clinical indicators which are incentivised in the GMS contract [14,18,21]. QALY estimates were based on lifetime QALY gains, on the assumption that appropriate treatment will continue beyond the current year. There are three ways in which to express health gain in a practice population. The 'maximum expected health gain' is the expected number of lives saved if all patients with the condition received indicated care. The 'maximum achievable health gain' is less, as it excludes those patients considered by their general practitioner to be not suitable for the intervention by exception reporting. For example, 13% of patients were excluded by exception reporting from receiving influenza immunization in 2005. The 'incentivised health gain' is the health gain at the performance threshold for maximum payment. This is even less as it is the expected number of lives saved when the target threshold for full payment is reached (for example full incentive payment is received in influenza immunization when 85% of eligible patients have received treatment).

### Analysis

We estimated health gain for an average sized practice of 6411 patients. The data for lives saved were expressed for an English population of 100,000 so we multiplied this by 0.06411 to reach the expected lives saved for a practice of size 6411. Data for QALYs gained were expressed for each patient treated, so we multiplied this figure by the number of patients that a practice of 6411 would have with that particular condition, using prevalence data at a national level from English practices in QOF [21].

To calculate the marginal incentive payment for a 1% increase in performance, the maximum total incentive payment was divided by the percentage difference between the upper and lower payment thresholds for payment. To calculate the marginal health gain from a one percentage point improvement in performance in each indicator the maximum achievable health gain was divided by 100. Indicator DM18 (influenza immunization in diabetes) in 2004 is used here as a worked example to demonstrate how marginal incentive payment and health gain was calculated. The health gain for full implementation of this indicator would be expected to yield 4.1 lives saved in an average practice (Table 1). However 13% of patients were exception reported leaving a maximum number of achievable lives saved of (1-0.13) × 4.1 which is 3.6 lives. A one percent increase in performance would yield 0.036 of a life saved. A one per cent increase in incentive for a practice performing within the upper and lower payment thresholds (25% to 85%) would be the maximum payment (£225) divided by the range (85%-25%) which is 225/60, or £3.75.

**Table 1:** Quality indicators, expected health gain, and payment for performance in 2004

Quality Indicator label	Indicator	Level of evidence^1^	Payment thresholds (%)	Payment range^2^	Maximum payment (£)	Marginal payment (£)^3^	E-reporting rate (%) 2005^4^	Maximum lives saved (n)	Achievable lives saved (n)	Incentivised lives saved (n)	Maximum QALYs gained (n)	Achievable QALYs gained (n)	Incentivised QALYs gained (n)
1	DM 18	c	25-85	60	225	3.75	13	4.1	3.6	3.0			

2	CHD 12	c	25-85	60	525	8.75	11	4	3.6	3.0			

3	BP 5	rct	25-70	45	4200	93.33	5	3.1	2.9	2.1	561	533	373

4	CHD 10	rct	25-50	25	525	21.00	25	2.9	2.2	1.1	427	320	160

5	Stroke 10	c	25-85	60	150	2.50	13	1.8	1.6	1.4			

6	DM 6	rct	25-50	25	1200	48.00	12	1.7	1.5	0.7			

8	COPD 8	c	25-85	60	450	7.50	11	1.6	1.4	1.2			

9	CHD 9	rct	25-90	65	525	8.08	4	1.6	1.5	1.4	2	2	2

11	CHD 8	rct	25-60	35	1200	34.29	10	1	0.9	0.5			

12	Stroke9	rct	25-90	65	300	4.62	5	1	1.0	0.9	11	10	9

13	DM 12	c	25-55	30	1275	42.50	8	0.9	0.8	0.5			

14	LVD 3	rct	25-70	45	750	16.67	7	0.8	0.7	0.5	5	5	3

15	CHD 6	rct	25-70	45	1425	31.67	4	0.7	0.7	0.5			

17	Asthma 5	c	25-70	45	450	10.00	3	0.6	0.6	0.4			

18	DM 7	c	25-85	60	825	13.75	6	0.4	0.4	0.3			

19	BP 3	c	25-90	65	750	11.54	1	0.3	0.3	0.3			

20	DM 15	rct	25-70	45	225	5.00	6	0.2	0.2	0.1	17	16	11

21	COPD 5	rct	25-90	65	450	6.92	2	0.2	0.2	0.2			

22	DM 4	c	25-90	65	375	5.77	3	0.1	0.1	0.1			

23	CHD 4	c	25-90	65	300	4.62	3	0.1	0.1	0.1			

24	CHD 11	rct	25-70	45	525	11.67	7	0.1	0.1	0.1	2	2	1

25	Stroke4	c	25-70	45	150	3.33	4	0.1	0.1	0.1			

27	CS1	c	25-80	55	825	15.00	5				71	67	54

28	DM8	c	25-90	65	375	5.77	6				115	108	97

All data for an average practice of 6411 patients, all figures rounded to whole numbers (except lives saved and marginal payment)

^1 ^rct = randomised controlled trial, c = cohort. ^2 ^% range of achievement between minimum and maximum payments

^3 ^£ incentive gained for a 1% increase in performance within payment range. ^4 ^E-reporting = exception reporting

For our primary analysis, we examined the relationship between the marginal increase in payment and health gain resulting from a one percent point improvement in performance for an average size general practice. For our secondary analysis we examined the relationship between the total payment and health gained at the performance target for maximum payment. Histograms showed that the data were not normally distributed; therefore a non-parametric Spearman rank correlation test was used in preference to a Pearson correlation test. A two-sided test was used to test the null hypothesis of no relationship between incentive payments and measures of health gain versus the alternative hypotheses of a positive or negative relationship. We tested this potential relationship for both the 2004 and 2006 versions of the GMS contract, and also for both health gain in terms of lives saved in one year and QALYs gained.

We conducted two sensitivity analyses. First we conducted a sensitivity analysis using the maximum number of achievable lives saved as practice performance often exceeded the upper threshold target for maximum payment. Second we included clinical indicators with evidence for health gain that were derived from randomised controlled trials only, excluding indicators with a lower strength of evidence. All tests were carried out in SPSS version 18.

## Results

### Primary analysis

Evidence for lives saved or QALYs gained was found for 28 indicators accounting for 41% of the total incentive payments. A full description of the clinical indicators is given in additional file 1. For an average practice population of 6411 the expected lives saved in one year by each indicator range from 0.1 to 4.1 (mean 1.6, standard deviation 1.2, Tables 1 &2). The expected lifetime QALYs gained ranges from 2 to 561 (mean 137, standard deviation 186). In our primary analysis in the 2004 QOF the correlation between achievable lives saved and incentive gained from a 1% increase in performance was not significant (Spearman's rho 0.216, p > 0.05, Table 3). The correlation between QALYs gained and incentive gained from a 1% increase in performance was also not significant (Spearman's rho 0.427, p > 0.05). In the 2006 QOF the correlation between achievable lives saved and incentive gained from a 1% increase in performance was not significant (Spearman's rho -0.026, p > 0.05). The correlation between QALYs gained and incentive gained from a 1% increase in performance was also not significant (Spearman's rho 0.368, p > 0.05).

**Table 2:** Quality indicators, expected health gain, and payment for performance in 2006

Quality Indicator label	Indicator	Level of evidence^1^	Payment thresholds (%)	Payment range^2^	Maximum payment (£)	Marginal payment (£)^3^	E-reporting rate (%) 2007^4^	Maximum lives saved (n)	Achievable lives saved (n)	Incentivised lives saved (n)	Maximum QALYs Gained (n)	Achievable QALYs Gained (n)	Incentivised QALYs gained (n)
1	DM 18	c	40-85	45	374	8.31	15	4.1	3.5	3.0			

2	CHD 12	c	40-90	50	872	17.44	13	4	3.5	3.1			

3	BP 5	rct	40-70	30	7102	236.73	4	3.1	3.0	2.1	561	539	377

4	CHD 10	rct	40-60	20	872	43.60	27	2.9	2.1	1.3	427	311	187

5	Stroke 10	c	40-85	45	249	5.53	15	1.8	1.5	1.3			

6	DM 20	rct	40-50	10	2118	211.80	10	1.7	1.5	0.8			

7	CKD3	c	40-70	30	1371	45.70	11	1.7	1.5	1.1			

8	COPD 8	c	40-85	45	748	16.62	13	1.6	1.4	1.2			

9	CHD 9	rct	40-90	50	872	17.44	3	1.6	1.5	1.4	2	2	2

10	AF3	rct	40-90	50	1869	37.38	4	2.9	2.8	2.5	183	176	158

11	CHD 8	rct	40-70	30	2118	70.60	9	1	0.9	0.6			

12	Stroke 12	rct	40-90	50	498	9.96	7	1	0.9	0.8	11	10	9

13	DM 12	c	40-60	20	2243	112.15	7	0.9	0.8	0.5			

14	LVD 3	rct	40-80	40	1246	31.15	8	0.8	0.7	0.6	5	5	4

15	CHD 6	rct	40-70	30	2367	78.90	3	0.7	0.7	0.5			

16	Smoking2	c	40-90	50	4361	87.22	1	0.7	0.7	0.6	14	14	12

18	DM 7	c	40-90	50	1371	27.42	5	0.4	0.4	0.3			

20	DM 15	rct	40-80	40	374	9.35	6	0.2	0.2	0.2	17	16	13

24	CHD 11	rct	40-80	40	872	21.80	8	0.1	0.1	0.1	2	2	1

26	CKD4	c	40-80	40	498	12.45	9				234	216	173

27	CS1	c	40-80	40	1371	34.28	6				71	67	53

28	DM 21	c	40-90	50	623	12.46	7				115	107	96

All data for an average practice of 6411 patients, all figures rounded to whole numbers (except lives saved and marginal payment)

^1 ^rct = randomised controlled trial, c = cohort. ^2 ^% range of achievement between minimum and maximum payments

^3 ^£ incentive gained for a 1% increase in performance within payment range. ^4 ^E-reporting = exception reporting

**Table 3:** Spearman correlations between measures of health gain and incentive payment

	Measure of health gain	Payment in 2004 QOF	Payment in 2006 QOF
Primary analysis	1% improvement in achievable lives saved and incentive gained	rho = 0.216 p = 0.334 n = 22	rho = -0.026 p = 0.917 n = 19
	
	1% increase in achievable QALYs and incentive gained	rho = 0.427 p = 0.252 n = 9	rho = 0.368 p = 0.240 n = 12

Secondary analysis	Lives saved to target and maximum incentive payment	rho = 0.183 p = 0.416 n = 22	rho = -0.186 p = 0.446 n = 19
	
	QALYs gained to target and maximum incentive payment	rho = 0.237 p = 0.539 n = 9	rho = 0.183 p = 0.568 n = 12

Sensitivity analysis 1	Maximum achievable lives saved and maximum incentive payment	rho = 0.213 p = 0.342 n = 22	rho = -0.087 p = 0.723 n = 19
	
	Maximum achievable QALYs gained and maximum incentive payment	rho = 0.238 p = 0.537 n = 9	rho = 0.184 p = 0.568 n = 12

	QOF = Quality and Outcomes Framework		

### Secondary and sensitivity analyses

**Figure 1:**
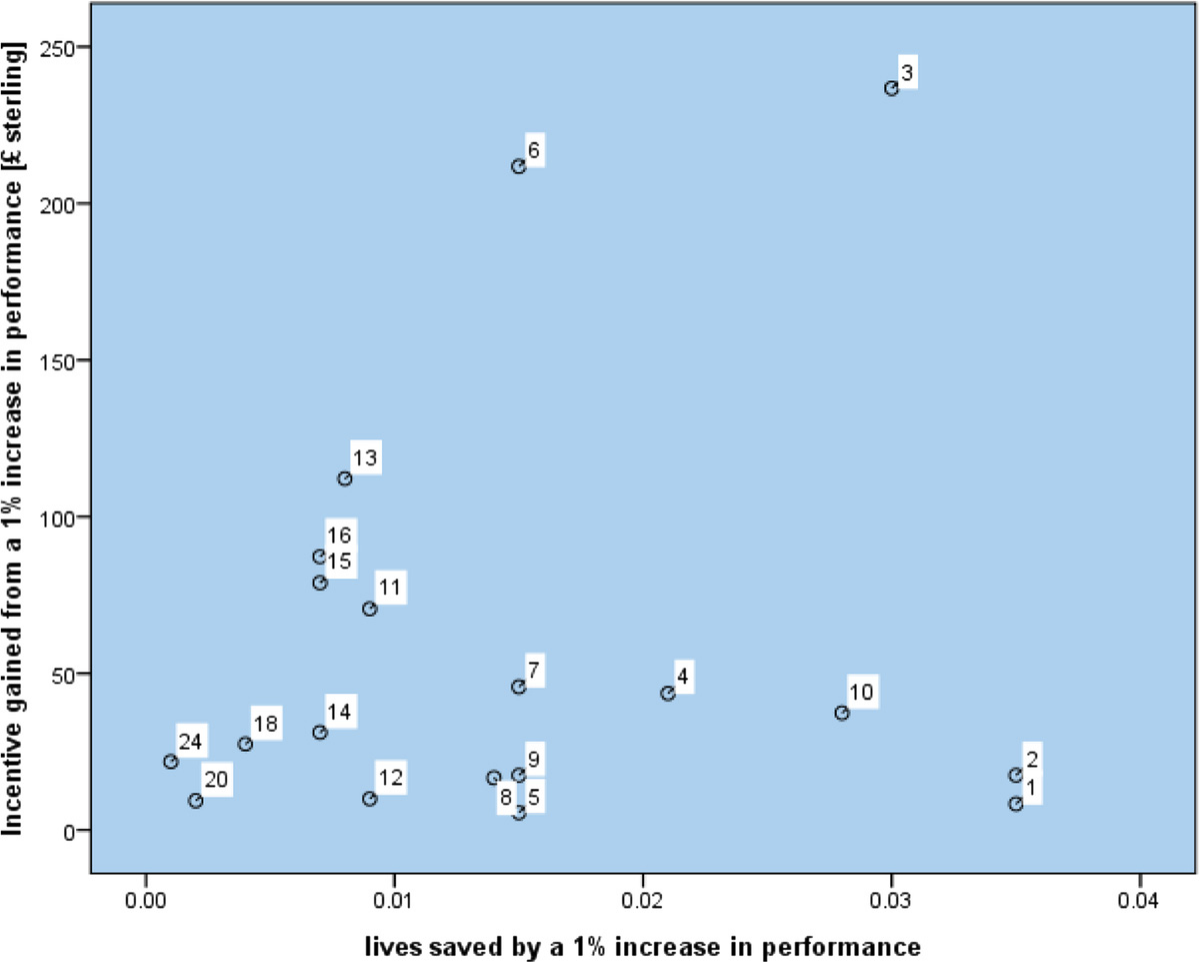
**Scatter plot of incentive gained and lives saved in quality indicators, 2006 contract**.

**Figure 2:**
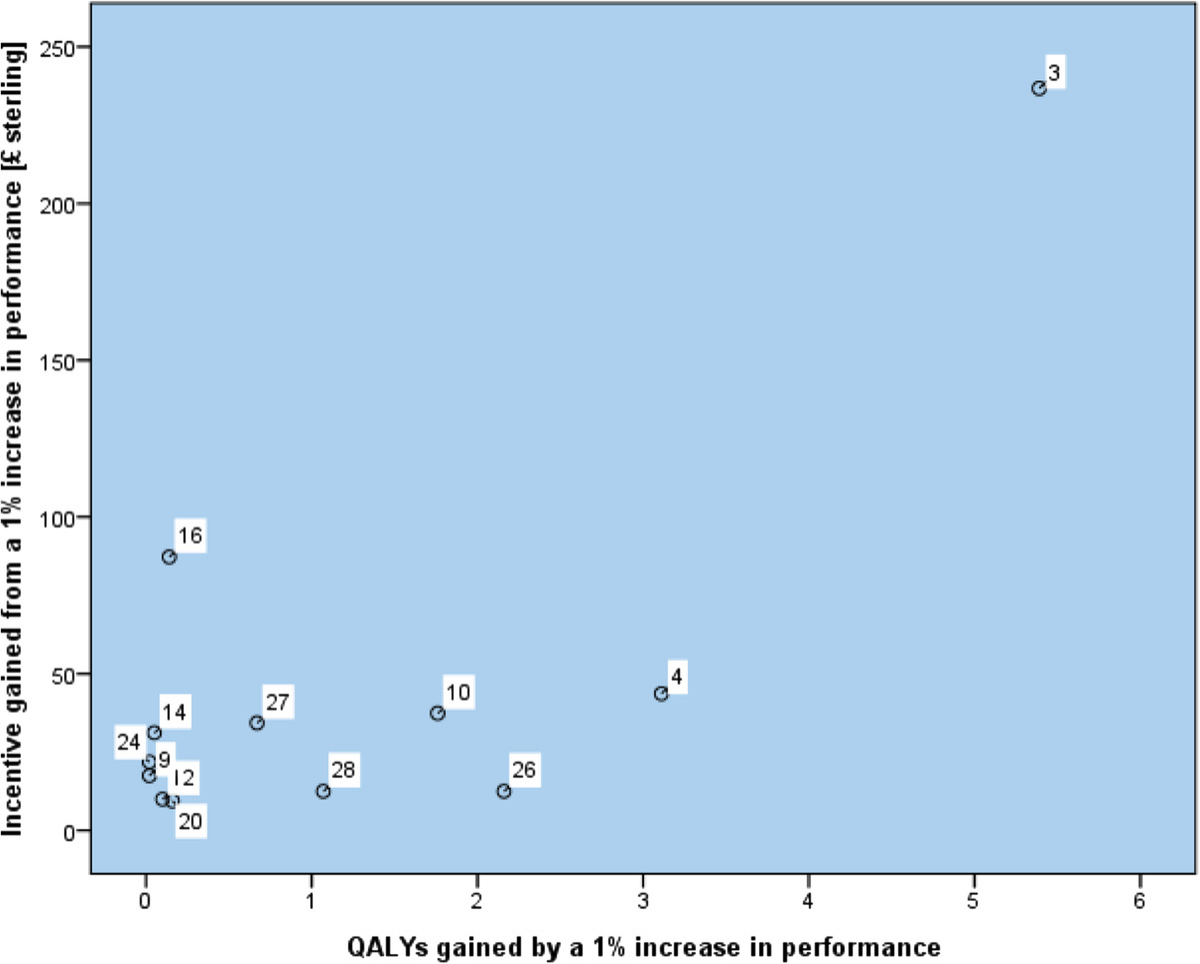
**Scatter plot of incentive and QALYs gained from a 1% increase in performance, 2006 contract**.

In our secondary analysis no associations were found between the size of financial payment for achievement of an indicator and the expected health gain at the performance threshold for maximum payment measured in lives saved or quality adjusted life years (Table 3). Our sensitivity analyses, firstly using 'maximum achievable health gain' rather than 'incentivised health gain' and secondly using evidence for health gain only from randomised controlled trials, did not substantially alter these findings (Tables 3 &4). Scatter plots presented in Figures 1 and 2 show one outlier, treatment of hypertension (label 3, indicator BP5). Reanalysis of the data after excluding this outlier also shows no significant associations. In summary no statistically significant associations were found between any measure of health gain and incentive gained in either the 2004 or 2006 version of the Quality and Outcomes Framework. We therefore cannot reject the null hypothesis of no relationship between incentive pay and health gain for all areas in both the 2004 and 2006 GMS contract.

**Table 4:** Sensitivity analysis 2, Spearman correlations between measures of health gain and incentive payment

Measure of health gain	2004 QOF payment (rcts only)	2006 QOF payment (rcts only)	2004 QOF payment (without BP5)	2006 QOF payment (without BP5)
1% improvement in achievable lives saved and incentive gained	Rho = 0.058 p = 0.111 n = 11	Rho = 0.058 p = 0.111 n = 11	Rho = 0.137 p = 0.553 n = 21	Rho = -0.147 p = 0.561 n = 18

1% increase in achievable QALYs and incentive gained	Rho = 0.450 p = 0.310 n = 7	Rho = 0.611 p = 0.108 n = 8	Rho = 0.180 p = 0.670 n = 8	Rho = 0.178 p = 0.601 n = 11

lives saved to target and maximum incentive payment	Rho = 0.381 p = 0.247 n = 11	Rho = 0.263 p = 0.434 n = 11	Rho = 0.095 p = 0.683 n = 21	Rho = -0.326 p = 0.186 n = 18

QALYs gained to target and maximum incentive payment	Rho = 0.148 p = 0.751 n = 7	Rho = 0.366 p = 0.373 n = 8	Rho = -0.098 p = 0.818 n = 8	Rho = -0.064 p = 0.851 n = 11

Maximum achievable lives saved and maximum incentive payment	Rho = 0.428 p = 0.189 n = 11	Rho = 0.412 p = 0.208 n = 11	Rho = 0.130 p = 0.572 n = 21	Rho = -0.223 p = 0.373 n = 18

Maximum achievable QALYs gained and incentive payment	Rho = 0.150 p = 0.749 n = 7	Rho = 0.368 p = 0.370 n = 8	Rho = -0.098 p = 0.817 n = 8	Rho = -0.065 p = 0.851 n = 11

## Discussion

The principal finding of this research is that there is no obvious relationship between the size of the financial incentive and health gain for indicators with available data on health gain in the 2004 or 2006 QOF for an average general practice. This is the case for both a marginal improvement in performance of 1% and also for maximum levels of performance. Some interventions (such as beta blockers in heart disease) receive a relatively low incentive compared with their expected health gain, whereas others (such as interventions for smoking cessation) receive a relatively high incentive compared with their expected health gain. Although the incentives were intended to reward general practice for both the volume and quality of the work done [1], our findings suggest that the incentives are not aligned with maximising health outcomes, which is an explicit aim of the Department of Health [2]. Many practices exceeded the threshold for maximum incentive payments in clinical indicators, and in these situations the GMS contract does not reward further improvement in the quality of care.

### Strengths and limitations

Strengths of this study include that robust and up-to-date measures of health gain were used which were computed specifically for the clinical indicators in the general practice contract, and included two different measures of health gain. The estimates of health gain are quite robust to measurement error, since all that is required for Spearman correlation tests in is that the estimates reflect the rankings of interventions by size of health gain. Sensitivity analyses excluding trials with lower levels of evidence and using maximum achievable health gain instead of incentivised health gain, show similar results.

Limitations include that we were only able to identify measures of health gain for a subset of 28 out of a total of 98 indicators in the 2004 and 2006 QOF including an additional area of cervical screening. However, these 28 are important indicators with measurable health outcomes and are all considered clinically important interventions, which account for an achievable 1,085 QALYs gained and 22 lives saved in one year for an average sized practice, and yield 41% of the maximum possible payment for clinical interventions. Of the clinical indicators that were not included in this study, a further 27 were processes which were related to achievement of these 28 indicators (additional file 1).

Not all QOF indicators may be mutually independent. For example a practice which has a successful influenza management system may be more likely to target all eligible patients for immunisation irrespective of which chronic disease they have. Furthermore if a patient has co morbidities such as diabetes and heart disease which include the same intervention, for example influenza immunisation (CHD 12 and DM 18) or hypertension control (CHD 6 and DM12), then there will be an inter dependency between these indicators in different disease domains. QOF interventions are selected by the NHS in agreement with the BMA and in consultation with stakeholder groups. The actions of these agencies at the primary care level may influence the priority that primary care gives to certain medical conditions in a number of ways in addition to inclusion in the QOF.

This research does not capture baseline performance, so our analysis is limited to examining pay for the level of performance, rather than for performance improvement. Our estimates of marginal incentive for a one percent point improvement in health gain are not affected by this problem, so long as baseline performance is below the target for maximum payment. We only considered the health benefits of the interventions in relation to the size of the QOF payments made. The costs of the interventions themselves and their effect on other health service costs have not been considered. Such costs would have to be considered if we were to evaluate the cost-effectiveness of the QOF. For example in another study it was found that two of the indicators considered actually reduced overall costs to the NHS (CHD 10- aspirin in heart disease and DM 15- ACE inhibitor drugs in diabetic renal disease) [21]. The net health impact of cost saving interventions could be higher than the average health impact we have estimated, since the resources saved can be used elsewhere for treating patients and delivering health gains. There will be a small number of individual practices with atypical populations where a closer national alignment of QOF incentives and expected health gain does not fit the health need for those particular practices. We have made the assumption that health gains are distributed evenly across all percentage increases in performance, which may not be the case.

### Comparison with previous research

Several studies have examined the expected health gain, effectiveness and cost effectiveness of QOF, and one examined the relationship between financial incentives and health gain [12,14,18,21]. The findings of this small study were similar to our study, finding no association between the size of the financial incentive and expected health gain [13].

### Implications

The main implication for policy makers is that the lack of an association between the size of the incentive and the expected health gain may risk skewing activity towards areas with high workload but relatively low benefit to health [3]. Other areas which receive little or no incentive may be relatively ignored [17,18]. Indications for further research include a systematic review of interventions across the spectrum of primary care to identify the evidence base in terms of QALYs to inform both the selection of new indicators and the relative size of financial incentives.

## Conclusions

When designing pay for performance schemes the size of achievable health gain to be expected from full implementation of an indicator should inform decisions both about which indicators to add and remove from future contract revisions, and about setting the relative size of the financial incentives. Where data on the size of the health gain for a clinical intervention do not exist, consideration should be given to commissioning research to produce the required information.

## Competing interests

Some of the data used was from a study funded by the Department of Health, to which the authors contributed. The authors work for this paper was independent of the DH. RC was a member of NICE advisory committees from 2002-2009, RF has contributed as an expert advisor to a NICE methods review, and NS currently sits on a NICE advisory committee.

## Authors' contributions

RF conceived the idea and designed the study, with supervision from RC and AH as part of his MD thesis. RC had a specific role in advising on the health economic and statistical aspects of the paper. RF wrote up the first draft of the study. All authors contributed to data analysis, contributed to revisions of the manuscript and approved the final draft. All authors read and approved the final manuscript.

## Pre-publication history

The pre-publication history for this paper can be accessed here:

http://www.biomedcentral.com/1472-6963/12/94/prepub

## Supplementary Material

Additional file 1 **Full description of clinical indicators**. Click here for file
