# Supplementary material for: Incentive payments are not related to expected health gain in the pay for performance scheme for UK primary care: cross-sectional analysis
Source: BMC Health Serv Res. 2012 Apr 16;12:94. doi: 10.1186/1472-6963-12-94 (PMC3365874; doi:10.1186/1472-6963-12-94)
Supplement: Additional file 1 — Full description of clinical indicators. [file 1472-6963-12-94-S1.DOC]

| **Indicator** | **label** | **Description** |
| --- | --- | --- |
| ***List A; Indicators included in the study*** | | |
| **DM 18** | **1** | The percentage of patients with diabetes who have had influenza immunisation in the preceding 1 September to 31 March |
| **CHD 12** | **2** | The percentage of patients with coronary heart disease who have had an influenza immunisation in the preceding 1 September to 31 March |
| **BP 5** | **3** | The percentage of patients with hypertension in whom the last blood pressure (measured in the last 9 months) is 150/90 or less |
| **CHD 10** | **4** | The percentage of patients with coronary heart disease who are currently treated with a beta blocker |
| **Stroke 10** | **5** | The percentage of patients with TIA or stroke who have had influenza immunisation in the preceding 1 September to 31 March |
| **DM 6** | **6** | The percentage of patients with diabetes in whom the last HbA1C is 7.4 or less (or equivalent test/reference range depending on local laboratory) in last 15 months |
| **CKD3** | **7** | The percentage of patients on the CKD register in whom the last blood pressure reading, measured in the previous 15 months, is 140/85 or less |
| **COPD 8** | **8** | The percentage of patients with COPD who have had influenza immunisation in the preceding 1 September to 31 March |
| **CHD 9** | **9** | The percentage of patients with coronary heart disease with a record in the last 15 months that aspirin, an alternative anti-platelet therapy, or an anti-coagulant is being taken (unless a contraindication or side-effects are recorded) |
| **AF3** | **10** | The percentage of patients with atrial fibrillation who are currently treated with anti-coagulant drug therapy or an anti-platelet therapy |
| **CHD 8** | **11** | The percentage of patients with coronary heart disease whose last measured total cholesterol (measured in last 15 months) is 5 mmol/l or less |
| **Stroke 9/12** | **12** | The percentage of patients with a stroke shown to be non haemorrhagic, or a history of TIA, who have a record that aspirin, an alternative anti-platelet therapy, or an anti-coagulant is being taken (unless a contraindication or side-effects are recorded) |
| **DM 12** | **13** | The percentage of patients with diabetes in whom the last blood pressure is 145/85 or less |
| **LVD 3** | **14** | The percentage of patients with a diagnosis of CHD and left ventricular dysfunction who are currently treated with ACE inhibitors (or A2 antagonists) |
| **CHD 6** | **15** | The percentage of patients with coronary heart disease in whom the last blood pressure reading (measured in the last 15 months) is 150/90 or less |
| **Smoking 2** | **16** | The percentage of patients with any or any combination of the following conditions: CHD, stroke or TIA, hypertension, diabetes, COPD or asthma who smoke whose notes contain a record that smoking cessation advice or referral to a specialist service, where available has been offered within the previous 15 months |
| **Asthma 5** | **17** | The percentage of patients with asthma who smoke, and whose notes contain a record that smoking cessation advice or referral to a specialist service, if available, has been offered within the last 15 months |
| **DM 7** | **18** | The percentage of patients with diabetes in whom the last HbA1C is 10 or less (or equivalent test/reference range depending on local laboratory) in last 15 months |
| **BP 3** | **19** | The percentage of patients with hypertension who smoke, whose notes contain a record that smoking cessation advice or referral to a specialist service, if available, has been offered at least once |
| **DM 15** | **20** | The percentage of patients with diabetes with proteinuria or micro-albuminuria who are treated with ACE inhibitors (or A2 antagonists) |
| **COPD 5** | **21** | The percentage of patients with COPD who smoke, whose notes contain a record that smoking cessation advice or referral to a specialist service has been offered in the past 15 months |
| **DM 4** | **22** | The percentage of patients with diabetes who smoke and whose notes contain a record that smoking cessation advice or referral to a specialist service, where available, has been offered in the last 15 months |
| **CHD 4** | **23** | The percentage of patients with coronary heart disease who smoke, whose notes contain a record that smoking cessation advice or referral to a specialist service, where available, has been offered within the last 15 months |
| **CHD 11** | **24** | The percentage of patients with a history of myocardial infarction (diagnosed after 1 April 2003) who are currently treated with an ACE inhibitor |
| **Stroke 4** | **25** | The percentage of patients with a history of TIA or stroke who smoke and whose notes contain a record that smoking cessation advice or referral to a specialist service, if available, has been offered in the last 15 months |
| **CKD 4** | **26** | The percentage of patients on the CKD register with hypertension, who are treated with an angiotensin converting enzyme inhibitor (ACE-I) or angiotensin receptor blocker (ARB) (unless a contraindication or side effects are recorded) |
| **CS 1** | **27** | The percentage of patients aged from 25 to 64 (in Scotland from 21 to 60) whose notes record that a cervical smear has been performed in the last five years |
| **DM 8/21** | **28** | The percentage of patients with diabetes who have a record of retinal screening in the previous 15 months |
| ***List B; Indicators related to those included in the study*** | | |
| **CHD 1** | related | The practice can produce a register of  patients with coronary heart disease |
| **CHD 3** | related | The percentage of patients with coronary heart disease whose notes record smoking status in the past 15 months |
| **CHD 5** | related | The percentage of patients with coronary heart disease whose notes have a record of blood pressure in the previous 15 months |
| **CHD 7** | related | The percentage of patients with coronary heart disease whose notes have a record of total cholesterol in the previous 15 months |
| **LVD/ HF 1** | related | The practice can produce a register of patients with heart failure |
| **LVD/ HF 2** | related | The percentage of patients with a diagnosis of heart failure (diagnosed after 1 April 2006) which has been confirmed by an echocardiogram or by specialist assessment |
| **Stroke 1** | related | The practice can produce a register of patients with Stroke or TIA |
| **Stroke 3** | Related | The percentage of patients with TIA or stroke who have a record of smoking status in the last 15 months, except those who have never smoked |
| **BP 1** | related | The practice can produce a register of patients with established hypertension |
| **BP 2** | related | The percentage of patients with hypertension who smoke, whose notes record smoking status at least once |
| **BP 4** | related | The percentage of patients with hypertension in whom there is a record of the blood pressure in the previous nine months |
| **DM 1/ 19** | related | The practice can produce a register of all patients aged 17 years and over with diabetes mellitus, which specifies whether the patient has Type 1 or Type 2 diabetes |
| **DM 3** | related | The percentage of patients with diabetes in whom there is a record of smoking status in the past 15 months |
| **DM 5** | related | The percentage of diabetic patients who have a record of HbA1c or equivalent in the previous 15 months |
| **DM 11** | related | The percentage of patients with diabetes who have a record of the blood pressure in the previous 15 months |
| **DM 13** | related | The percentage of patients with diabetes who have a record of micro-albuminuria testing in the previous 15 months (exception reporting for patients with proteinuria) |
| **COPD 1** | related | The practice can produce a register of patients with COPD |
| **COPD 4** | related | The percentage of patients with COPD in whom there is a record of smoking status in the past 15 months |
| **COPD 5** | related | The percentage of patients with COPD who smoke, whose notes contain a record of smoking cessation advice in the past 15 months |
| **Asthma 1** | related | The practice can produce a register of patients with asthma, excluding patients with asthma who have been prescribed no asthma-related drugs in the previous twelve months |
| **Asthma 3** | related | The percentage of patients with asthma between the ages of 14 and 19 in whom there is a record of smoking status in the previous 15 months |
| **Asthma 4** | related | The percentage of patients aged 20 or over with asthma whose notes record smoking status in the past 15 months |
| **CKD 1** | related | The practice can produce a register of patients aged 18 years and over with CKD (US National Kidney Foundation: Stage 3 to 5 CKD) |
| **CKD 2** | related | The percentage of patients on the CKD register whose notes have a record of blood pressure in the previous 15 months |
| **AF 1** | related | The practice can produce a register of patients with atrial fibrillation |
| **AF 2** | related | The percentage of patients with atrial fibrillation diagnosed after 1 April 2006 with ECG or specialist confirmed diagnosis |
| **Smoking 1** | related | The percentage of patients with any or any combination of the following conditions: coronary heart disease, stroke or TIA, hypertension, diabetes, COPD or asthma whose notes record smoking status in the previous 15 months. Except those who have never smoked where smoking status need only be recorded once since diagnosis |
| ***List C; Indicators excluded from the study*** | | |
| **CHD 2** | **excluded** | The percentage of patients with newly diagnosed  angina (diagnosed after 1 April 2003) who are referred  for exercise testing and/or specialist assessment |
| **Stroke 2** | **excluded** | The percentage of new patients with presumptive stroke who have been referred for a CT/ MRI scan |
| **Stroke 5** | **excluded** | The percentage of patients with TIA or stroke who have a record of blood pressure in the notes in the preceding 15 months |
| **Stroke 6** | **excluded** | The percentage of patients with a history of TIA or stroke in whom the last blood pressure reading (measured in the previous 15 months) is 150/90 or less |
| **Stroke 7** | **excluded** | The percentage of patients with TIA or stroke who have a record of total cholesterol in the last 15 months |
| **Stroke 8** | **excluded** | The percentage of patients with TIA or stroke whose last measured total cholesterol (measured in the previous 15 months) is 5 mmol/l or less |
| **Stroke 11** | **excluded** | The percentage of new patients with a stroke who have been referred for further investigation |
| **DM 2** | **excluded** | The percentage of patients with diabetes whose notes record BMI in the previous 15 months |
| **DM 8/ 21** | **excluded** | The percentage of patients with diabetes who have a record of retinal screening in the previous 15 months |
| **DM 9** | **excluded** | The percentage of patients with diabetes with a record of the presence or absence of peripheral pulses in the previous 15 months |
| **DM 10** | **excluded** | The percentage of patients with diabetes with a record of neuropathy testing in the previous 15 months |
| **DM 14/ 22** | **excluded** | The percentage of patients with diabetes who have a record of estimated glomerular filtration rate (eGFR) or serum creatinine testing in the previous 15 months |
| **DM 16** | **excluded** | The percentage of patients with diabetes who have a record of total cholesterol in the previous 15 months |
| **DM 17** | **excluded** | The percentage of patients with diabetes whose last measured total cholesterol within previous 15 months is 5 mmol/l or less |
| **COPD 2/3/9** | **excluded** | The percentage of all patients with COPD in whom diagnosis has been confirmed by spirometry including reversibility testing |
| **COPD 6/ 10** | **excluded** | . The percentage of patients with COPD with a record of FeV1 in the previous 15 months |
| **COPD 7/11** | **excluded** | The percentage of patients with COPD receiving inhaled treatment in whom there is a record that inhaler technique has been checked in theprevious 15 months |
| **Epilepsy 1/5** | **excluded** | The practice can produce a register of patients aged 18 and over receiving drug treatment for epilepsy |
| **Epilepsy 2/6** | **excluded** | The percentage of patients age 18 and over on drug treatment for epilepsy who have a record of seizure frequency in the previous 15 months |
| **Epilepsy 3/7** | **excluded** | The percentage of patients age 18 and over 4 40–90% on drug treatment for epilepsy who have a record of medication review involving the patient and/or carer in the previous 15 months |
| **Epilepsy 4/8** | **excluded** | The percentage of patients age 18 and over on drug treatment for epilepsy who have been seizure free for the last 12 months recorded in the previous 15 months |
| **Thyroid 1** | **excluded** | The practice can produce a register of patients with hypothyroidism |
| **Thyroid 2** | **excluded** | The percentage of patients with hypothyroidism with thyroid function tests recorded in the previous 15 months |
| **Cancer 1** | **excluded** | The practice can produce a register of all cancer patients defined as a ‘register of patients with a diagnosis of cancer excluding non-melanotic skin cancers from 1 April 2003’ |
| **Cancer 2/3** | **excluded** | The percentage of patients with cancer, diagnosed within the last 18 months who have a patient review recorded as occurring within six months of the practice receiving confirmation of the diagnosis |
| **PC 1** | **excluded** | The practice has a complete register available of all patients in need of palliative care/support |
| **PC 2:** | **excluded** | The practice has regular (at least three monthly) multidisciplinary case review meetings where all patients on the palliative care register are discussed |
| **MH 1/8** | **excluded** | The practice can produce a register of people with schizophrenia, bipolar disorder and other psychoses |
| **MH 2/9** | **excluded** | The percentage of patients with schizophrenia, bipolar affective disorder and other psychoses with a review recorded in the preceding 15 months. In the review there should be evidence that the patient has been offered routine health promotion and prevention advice appropriate to their age, gender and health status |
| **MH 3** | **excluded** | The percentage of patients on lithium therapy with a record of lithium levels checked within the previous 6 months |
| **MH 4** | **excluded** | The percentage of patients on lithium therapy with a record of serum creatinine and TSH in the preceding 15 months |
| **MH 5** | **excluded** | The percentage of patients on lithium therapy with a record of lithium levels in the therapeutic range within the previous six months |
| **MH 6** | **excluded** | The percentage of patients on the register who have a comprehensive care plan documented in the records agreed between individuals, their family and/or carers as appropriate |
| **MH 7** | **excluded** | The percentage of patients with schizophrenia, bipolar affective disorder and other psychoses who do not attend the practice for their annual review who are identified and followed up by the practice team  within 14 days of non-attendance |
| **Asthma 2/8** | **excluded** | The percentage of patients aged eight and over diagnosed as having asthma from 1 April 2006 with measures of variability or reversibility |
| **Asthma 6** | **excluded** | The percentage of patients with asthma who have had an asthma review in the previous 15 months |
| **Asthma 7** | **excluded** | The percentage of patients aged 16 years and over who have had an influenza immunisation in the preceding 1 September to 31 March |
| **DEM 1** | **excluded** | The practice can produce a register of patients diagnosed with dementia |
| **DEM 2** | **excluded** | The percentage of patients diagnosed with dementia whose care has been reviewed in the previous 15 months |
| **DEP 1** | **excluded** | The percentage of patients on the diabetes register and/or the CHD register for whom case finding for depression has been undertaken on one occasion during the previous 15 months using two standard screening questions |
| **DEP 2** | **excluded** | In those patients with a new diagnosis of depression, recorded between the preceding 1 April to 31 March, the percentage of patients who have had an assessment of severity at the outset of treatment using  an assessment tool validated for use in primary care |
| **Obesity 1** | **excluded** | The practice can produce a register of patients aged 16 and over with a BMI greater than or equal to 30 in the previous 15 months |
| **Learning disability** | **excluded** | The practice can produce a register of patients with 4  learning disabilities |
| **AF: atrial fibrillation, BP: Blood pressure (hypertension), CHD: coronary heart disease, CKD: chronic kidney disease, COPD: chronic obstructive airways disease, , CS: cervical screening, DEM: dementia, DEP: depression, DM: diabetes, HF: heart failure, LVD: heart failure, MH: mental health, PC: palliative care,** | | |
